# Over the Counter Availability of Antituberculosis Drugs in Tbilisi, Georgia in the Setting of a High Prevalence of MDR-TB

**DOI:** 10.1155/2009/513609

**Published:** 2009-06-11

**Authors:** Ketevan Kobaidze, Archil Salakaia, Henry M. Blumberg

**Affiliations:** ^1^Division of General Medicine, Department of Medicine, Emory University School of Medicine, Atlanta, GA 30308, USA; ^2^National Center for Tuberculosis and Lung Diseases, Tbilisi 0101, Georgia; ^3^Division of Infectious Diseases, Department of Medicine, Emory University School of Medicine, Atlanta, GA 30303, USA

## Abstract

Georgia, a country of 4.5 million people, has a high incidence of tuberculosis (TB) including drug resistant cases. Easy access and inappropriate use of anti-TB drugs are risk factors for further development of multidrug resistant (MDR)-TB. We carried out an investigation to assess the availability of over the counter anti-TB agents in pharmacies in Tbilisi. During February 2006, 15 pharmacies were randomly selected and the pharmacist at each store was interviewed. We found that all anti-TB medications stocked by these pharmacies were available and sold without a prescription. All 15 pharmacies sold isoniazid, rifampicin, and streptomycin; 13 (87%) of 15 pharmacies also sold pyrazinamide, ethambutol. Second line anti-TB drugs such as amikacin and kanamycin (injectable agents) and older fluoroquinolones (ofloxacin and ciprofloxacin) were available at 13 pharmacies while newer generation fluoroquinolones were less available(3 sold leovofloxacin, none sold moxifloxacin). The ease access and availability of anti-TB agents is of a great concern given the high prevalence of TB including MDR-TB in Georgia. The potential for misuse of these anti-TB drugs can lead to the development of further drug resistance. These drugs should only be available by prescription in order to reduce the chance of amplifying drug resistance.

## 1. Background

Tuberculosis (TB) is an enormous global public health problem with approximately 9 million new cases and 2 million deaths due to the disease each year [1]. The worldwide emergence of drug-resistant TB has greatly threatened global TB control efforts. Multidrug resistant TB (MDR-TB) defined as resistance to at least both isoniazid and rifampin is now widespread [2]. MDR-TB is particularly challenging to treat and to be associated with high morbidity and mortality. Recent reports have highlighted the emergence of extensively drug resistant TB (XDR-TB, defined as MDR strains which are also resistant to a fluoroquinolone and an injectable drug such as amikacin, kanamycin, or capreomycin) which is virtually untreatable [3, 4]. The World Health Organization (WHO) global surveillance for anti–tuberculosis drug resistance has found that the highest prevalence of drug resistant TB including MDR-TB is in former Soviet republics [2]. 

Georgia, a country of 4.5 million people, located in the Caucuses has a high incidence of TB as well as a high prevalence of drug resistant TB. In 2005, a total of 6448 TB cases were reported in Georgia; the incidence and prevalence of TB was 97 cases and 147 cases per 100 000 population, respectively [5]. A recent population-based drug resistant survey found that the prevalence of MDR-TB in Georgia was ~7% among newly diagnosed patients and 27% among previously treated patients [6]. 

Inappropriate use of anti-tuberculosis drugs, inappropriate treatment regimens, failure to ensure adherence by the delivery of therapy by directly observed therapy (part of the WHO-recommended DOTS strategy), interrupted supplies of drugs, and poor quality drugs are risk factors for the emergence of MDR-TB [7]. In Georgia, the National Tuberculosis Program (NTP) endorsed the Directly Observed Therapy, Short-Course (DOTS) strategy in 1997, but it has only been in the past few years that substantial areas of Georgia have had DOTS implementation. Inappropriate use of anti-tuberculosis antibiotics is associated with the emergence of resistance. Several authors reported interesting data about over-the-counter availability of anti-tuberculosis medications in private pharmacies in Bolivia, Vietnam, Nepal, and India [8–11]. Over-the-counter antibiotics including anti-tuberculosis agents are available at pharmacies in Georgia as is the case in most other resource limited areas. Easy access and inappropriate use of these drugs are risk factors for further development of drug resistant–TB in Georgia. Given the lack of data on which agents are available over the counter, we carried out an investigation to assess availability, price, and source of anti-TB agents available over the counter in pharmacies in Tbilisi, Georgia.

## 2. Methods

A questionnaire was developed to survey the over-the-counter availability of anti-TB agents at pharmacies in Tbilisi, Georgia. During February 2006, 15 pharmacies in Tbilisi, Georgia were randomly selected for assessment from pharmacies listed in the Tbilisi “Yellow Pages” directory. The pharmacist at each store was interviewed by one of the authors (KK) and provided the current retail price of each anti-TB antibiotic. They were also asked their opinion on future plans for sales of these drugs. Data from the completed questionnaires were entered into Epi-Info (CDC, Atlanta, Ga, USA) which was used to perform analyses.

## 3. Results

All anti-TB medications stocked by these 15 pharmacies were available and sold without a prescription. The availability, origin, and price are summarized in Table 1. First-line drugs were readily available in pharmacies. All 15 pharmacies sold isoniazid, rifampin, and streptomycin, and 13 (87%) of 15 pharmacies also stocked and sold pyrazinamide (PZA) and ethambutol. A number of second-line drugs were also available over the counter. Thirteen (87%) of 15 pharmacies sold the injectable agents amikacin and kanamycin which are preferred agents used in the treatment of MDR-TB. Fluoroquinolones were available at most pharmacies. Ofloxacin and ciprofloxacin were available at 13 (87%) pharmacies while newer-generation fluoroquinolones were less available; levofloxacin was sold at 3 (20%) pharmacies and none carried gatifloxacin or moxifloxacin. Other second-line drugs including para-aminosalicylic acid (PAS), cycloserine, and capreomycin were not available at any of the pharmacies. The mean price for these drugs is shown in Table 1. Prices for the individual products were remarkably similar among the pharmacy shops (data not shown). However, there were noticeable differences in prices between brand manufactured in Europe or the United States which were higher in price than the same drug manufactured in Russia or India (Table 1).

## 4. Discussion/Conclusions

We found that anti-TB drugs were widely available over the counter without a prescription at pharmacies in Tbilisi, the capital of Georgia. This included the universal availability of isoniazid and rifampin, the two most important first line agents as well as most other first line agents. In addition, a number of the most important second-line agents used to treat MDR-TB including injectable agents (amikacin and kanamycin) and fluoroquinolones were also available at most pharmacies. The ease of availability of these agents is of great concern given the high prevalence of TB including MDR-TB in Georgia and the potential for misuse of these antituberculosis drugs which can spawn further drug resistance (including the development of XDR-TB due to misuse of second-line drugs). At the time of the survey, second-line drugs and treatment of MDR-TB were not available through the Georgian National Tuberculosis Program (NTP). In 2007, the Georgian NTP began pilot projects for the treatment of MDR-TB and received approval from the WHO Green Light Committee (GLC) to procure second-line drugs which is being supported through a grant from the Global Fund to Fight AIDS, Tuberculosis, and Malaria. Given the gravity of TB and drug resistant TB in Georgia, education of policy makers as well as public health advocates, physicians, pharmacists, and the public about the potential impact and misuse of anti-tuberculosis drugs which are available over the counter without a prescription is critical if a national policy is to be established to restrict the sale of over the counter anti-TB drugs. Anti-tuberculosis drugs should only be received by directly observed therapy through the National Tuberculosis Program. When drugs which can be used to treat TB are dispensed by pharmacists (e.g., second-line drugs such as fluroquinolones or amikacin which are also used to treat bacterial infections), these drugs should only be available by prescription in order to reduce the chance of amplifying drug resistance through inappropriate use of these agents. 

## Figures and Tables

**Table 1:** Availability of Antituberculosis Antibiotics at 15 Pharmacies in Tblisi, Georgia Over the Counter without a Prescription.

Product (*n* = number of pharmacies stocking product)	Dose	Sale unit	Country of manufacture	Mean Price per sale Unit	Mean price per daily dose for a 70 kg adult (USD)
Isoniazid (*n* = 15)	300 mg	100 pills	Russia	2.06 L/$1.12	$0.01
Rifampicin (*n* = 15)	300 mg	10 pills	Italy	3.00 L/$1.63	$0.33
			Russia	2.00 L/$1.08	$0.22
			Germany	2.50 L/$1.63	$0.33
			India	2.00 L/$1.08	$0.22
Ethambutol (*n* = 13)	400 mg	50 pills	Russia	5.00 L/$2.72	$0.16
		10 pills	Germany	2.00 L/$1.09	$0.33
		50 pills	Ukraine	5.00 L/$2.72	$0.16
Pyrazinamide(PZA) (*n* = 13)	500 mg	100 pills	Slovenia	20.00 L/$10.87	$0.33
			Germany	20.00 L/$10.87	$0.33
Streptomycin (*n* = 15)	1 gram	1 amp	Russia	0.19 L/$0.10	$0.10
			Ukraine	0.21 L/$0.11	$0.11
Amikacin (*n* = 13)	500 mg	1 amp	Italy	9.00 L/$4.89	$9.78
	250 mg	1 amp	Bulgaria	1.80 L/$0.98	$3.92
	500 mg	1 amp	USA	9.00 L/$4.89	$9.78
Kanamycin (*n* = 13)	1 gram	1 amp	Russia	0.30 L/$0.16	$0.16
			Ukraine	0.30 L/$0.16	$0.16
Ciprofloxacin (*n* = 13)	500 mg	10 pills	Germany	51.00 L/$27.72	$8.31
		10 pills	India	1.50 L/$0.82	$0.24
		10 pills	Bulgaria	3.80 L/$2.07	$0.62

L – Lari, Georgian currency, at the time of the interview $1 USD = 1.84L. Capreomycin, moxifloxacin, gatifloxacin, para-aminosalicylic acid (PAS), ethionamide, and cycloserine were not available at any of the pharmacies.
